# Supplementary material for: Comparative multiomics analysis of cell physiological state after culture in a basket bioreactor
Source: Sci Rep. 2022 Nov 23;12:20161. doi: 10.1038/s41598-022-24687-4 (PMC9686226; doi:10.1038/s41598-022-24687-4)

[illegible]3666038.1:44160457:44160518:+@NW_023666038.1:44160457:44160569:+@NW_023666038.1:44172267:4417[illegible]

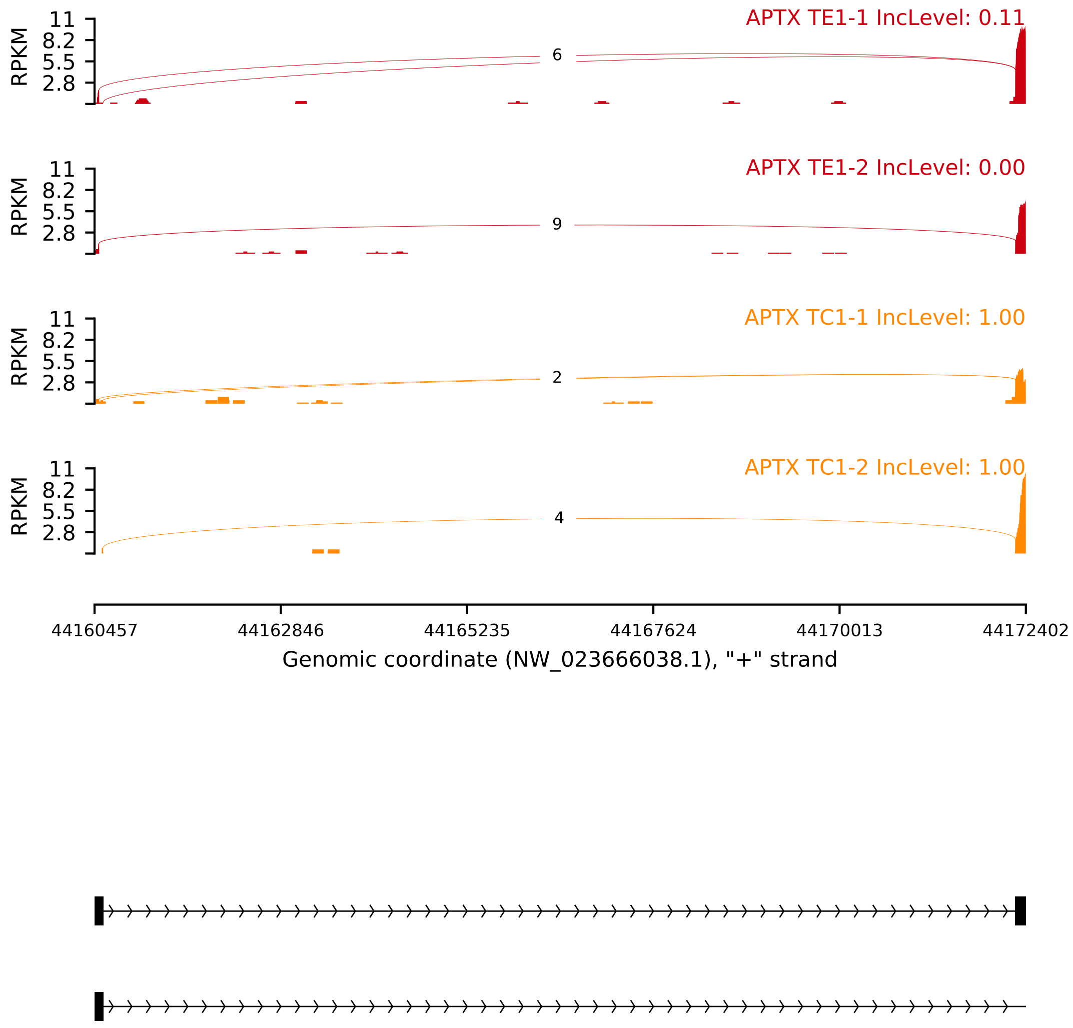

Supplement: Supplementary file 1 — Supplementary Information 1. [file 41598_2022_24687_MOESM1_ESM.zip › raw data/Transcriptomic raw data/7.AS/2.dasplot/TE1vsTC1/A5SS/1_APTX_NW_023666038.1_44160457_44160518_+@NW_023666038.1_44160457_44160569_+@NW_023666038.1_44172267_44172403_+.pdf]
